# Acetaminophen versus Ibuprofen for the management of hemodynamically significant patent ductus arteriosus in very low birth weight infants: randomized trial

**DOI:** 10.1016/j.jped.2026.101580

**Published:** 2026-08-07

**Authors:** Aldo Bancalari, Horacio Contreras, Valentina Díaz, Jimena Giaconi, Ivonne Henriquez, Gloria Pérez, Andrés Marinovic

**Affiliations:** a University of Concepción, School of Medicine, Department of Pediatrics, Concepción, Chile; b Guillermo Grant Benavente Hospital, Neonatology Service, Concepción, Chile

**Keywords:** Acetaminophen, Ibuprofen, PDA, Preterm infant

## Abstract

**Objective:**

The objective was to evaluate the efficacy and safety of Acetaminophen versus Ibuprofen in the treatment of hemodynamically significant patent ductus arteriosus.

**Method:**

A randomized, partially blinded controlled study was conducted in very low birth weight infants. Diagnostic criteria included a ductal diameter of 1.5 millimeters or greater, left cavities dilation or a left atrium to aortic root ratio greater than 1.4, and retrograde flow. Preterm infants were randomized into intravenous Acetaminophen or Ibuprofen treatment groups. Ductal closure was evaluated at the end of the third treatment day. A blood count and renal and liver function tests were done before and after treatment.

**Results:**

120 preterm infants were enrolled; 60 were randomized to each treatment group. The average pre-treatment ductal diameter was similar between both groups. Mean weight and gestational age were 1145 grams and 28.3 weeks for Acetaminophen, and 1096 grams and 27.6 weeks for Ibuprofen. The ductal closure rate after the first treatment course was 66.7% in the Acetaminophen group and 63.3% in the Ibuprofen group (*p* = 0.70), increasing to 88.3% and 86.7%, respectively, after the second course (*p* = 0.78). In the Acetaminophen group, the need for surgical closure was 10% and in the Ibuprofen group 11.7% (*p* = 0.77). No significant differences were observed in laboratory tests before and after treatment.

**Conclusion:**

Acetaminophen may be considered a safe and effective alternative, with no differences in side effects, to ibuprofen for treating hemodynamically significant patent ductus arteriosus in very low birth weight premature infants.

## Introduction

Hemodynamically significant Patent Ductus Arteriosus (hs-PDA) is one of the most challenging problems in neonatal intensive care. Currently, there is a controversy in the treatment of ductus regarding the timing and the best pharmacological alternative in terms of efficacy and minimal side effects [1].

The management of hs-PDA in preterm infants remains controversial, and recent evidence does not support routine early pharmacological closure in all cases. Contemporary trials and meta-analyses suggest that expectant management may be non-inferior, and in some analyses associated with better morbidity and mortality outcomes, compared with active treatment during the first two weeks of life [2,3]. However, when pharmacological closure is considered clinically necessary because of significant ductal shunting, pulmonary overcirculation, or systemic hypoperfusion, ibuprofen is commonly recommended as the preferred pharmacological option, while acetaminophen has emerged as a potential alternative with comparable closure rates and a different safety profile [4]

Hemodynamically significant patent ductus arteriosus is a frequent morbidity in very low birth weight newborns (VLBW), with an incidence close to 30% in these infants [5]. At lower gestational age and birth weight, the incidence increases, being greater than 50% in extremely low birthweight newborns (< 1000 g) [6].

The left-to-right shunting through an hs-PDA has been associated with an increase in several comorbidities, such as pulmonary hemorrhage, renal failure, necrotizing enterocolitis, bronchopulmonary dysplasia, and intraventricular hemorrhage [6,7]. Timely closure of the hs-PDA may be important to reduce these complications, and thus increase intact survival, especially in extremely low birth weight infants [6,7].

Currently, the first therapeutic option for closure of a patent ductus arteriosus in VLBW infants is the use of cyclooxygenase inhibitors (NSAIDs) such as ibuprofen or indomethacin, with an estimated success rate of 60 to 80% [5,8]. However, its use is not exempt from side effects, such as peripheral vasoconstriction, decreased platelet count, gastrointestinal bleeding, hyperbilirubinemia, and renal failure [[9], [10], [11], [12]].

For this reason, it is important to search for and use new pharmacological treatment options. The use of intravenous acetaminophen in the closure of hs-PDA has been studied in recent years, as a therapeutic alternative in its pharmacological closure, with satisfactory results and fewer adverse effects compared to cyclooxygenase inhibitors [5,6,11,13].

Acetaminophen promotes ductal closure by decreasing prostaglandin synthesis, particularly prostaglandin E2, which is one of the main mediators maintaining ductal patency in preterm infants. Unlike indomethacin and ibuprofen, which are non-selective cyclooxygenase inhibitors acting primarily at the cyclooxygenase site of prostaglandin H2 synthase, acetaminophen appears to act mainly at the peroxidase site of the same enzyme complex, reducing the conversion of arachidonic acid into prostaglandin precursors [8]. This difference may explain its weaker peripheral anti-inflammatory activity and its more limited effect on systemic vascular tone compared with NSAIDs. Consequently, acetaminophen is not associated with the same degree of systemic, renal, mesenteric, or cerebral vasoconstriction described with indomethacin and ibuprofen, potentially reducing adverse effects [5,[9], [10], [11]].

In this study, the intravenous route was selected to ensure reliable drug delivery and more predictable systemic exposure in very low birth weight preterm infants, in whom enteral administration may be limited by feeding intolerance, delayed gastric emptying, fluid restriction, regurgitation, or concerns regarding gastrointestinal morbidity. This route also allowed a more standardized comparison between acetaminophen and ibuprofen, reducing variability related to gastrointestinal absorption and thereby aligning with the study objective of evaluating efficacy and safety in the pharmacological treatment of hemodynamically significant patent ductus arteriosus.

In this regard, acetaminophen has shown an adequate safety profile and does not appear to compromise cerebral perfusion or cerebral oxygenation in preterm infants treated for patent ductus arteriosus [14]. For this reason, it has been used as an alternative in patients with contraindications to NSAIDs, such as renal impairment, gastrointestinal complications, thrombocytopenia, or increased bleeding risk [6]. Although potential hepatotoxicity related to acetaminophen metabolism has been postulated, available neonatal studies have not shown significant changes in transaminase levels after treatment [5,13,15].

Based on the above, it is important to establish a new pharmacological therapeutic alternative, with similar efficacy to the standard treatment with NSAIDs [[16], [17], [18]]. but with fewer side effects and contraindications [7,13,19].

The hypothesis of the present study was that acetaminophen has an effectiveness similar to ibuprofen in closing hs-PDA, but with fewer side effects.

According to our knowledge, there are no available studies that compare the efficacy and safety of acetaminophen versus ibuprofen as treatment of hs-PDA in Latin America.

The objective was to evaluate the efficacy and safety of intravenous acetaminophen versus intravenous ibuprofen in the treatment of hemodynamically significant patent ductus arteriosus in very low birth weight preterm infants.

## Methods

This was a randomized prospective study in very low birth weight preterm newborns < 32 weeks GA and/or ≤ 1500 g with an echocardiographic diagnosis of hs-PDA, performed during the first 2 weeks of life, between January 2017 and December 2019.

The diagnosis of hs-PDA was made by echocardiography using two-dimensional color Doppler (Vivid-i, GE Healthcare), carried out by a pediatric cardiologist who was blinded to the study treatment groups. The treating medical team was also unaware of the indicated pharmacological treatment.

The echocardiographic criteria to define hs-PDA were: ductal diameter ≥1.5 mm or equal to or greater than that of the left pulmonary artery (LPA), measured in the right parasternal short axis; left cavities dilation or left atrial-aortic ratio (LA/Ao) > 1.4, measured in the left parasternal long axis; retrograde flow measured in the descending aorta, celiac or mesenteric artery [20].

Patients who met the inclusion criteria were randomized to the acetaminophen or ibuprofen group, using an opaque sealed envelope, sequentially numbered. The acetaminophen dose was 15 mg/kg every 6 h for 3 days intravenously. (Paracetamol Kabi™ preparation (10 mg/mL), Fresenius Kabi Laboratory). The drug was administered IV for one hour or less. The ibuprofen dose was 10 mg/kg on the first day and then 5 mg/kg on the following two days (every 24 h). (Ibuprofen Pedea ™ preparation (5 mg/mL), Orphan Europe Laboratory). Ibuprofen was diluted in a solution of sodium chloride 0.9%, resulting in a concentration of 1 mg/mL. This drug was administered IV for one hour or less.

The difference in dosing intervals (acetaminophen every 6 h versus ibuprofen every 24 h) reflects the specific pharmacokinetic profiles and the internationally established therapeutic protocols for each drug. Utilizing their respective standard dosing regimens ensures that optimal plasma concentrations are maintained for both medications. Therefore, comparing these drugs under their optimal, standard-of-care conditions preserves the scientific and clinical comparability of the trial.

Inclusion criteria were: preterm newborns between 25^+0^and 31^+6^ weeks of gestational age and/or ≤ 1500 g of birth weight; echocardiographic diagnosis of hs-PDA within the first two weeks after birth; and admitted to the neonatal intensive care unit of Guillermo Grant Benavente Hospital.

Exclusion criteria were: major congenital malformations, fetal hydrops, complex or ductus dependent congenital heart disease; septic shock; grade III and IV intracranial hemorrhage; renal failure with urine output < 1 cc/kg/h in the previous 24 h; serum creatinine concentration > 1.5 mg/dL; platelet count < 50,000 mm³; major bleeding (defined as the presence of massive hematuria and/or blood in tracheal aspirate), hyperbilirubinemia in the range of exchange transfusion; confirmed necrotizing enterocolitis (Bell stage > II); liver dysfunction defined as elevation of liver enzymes alanine amino transferase (ALT) and aspartate amino transferase (AST) over two times the upper limit of normal range (ALT 6-50 U/L and AST 35-140 U/L) [21].

The criteria for suspending treatment with ibuprofen were: renal failure with serum creatinine greater than 2.5 mg/dL; evidence of bleeding; intestinal perforation; confirmed necrotizing enterocolitis, and for acetaminophen, evidence of acute liver failure with abrupt changes in coagulation tests not explained by consumptive coagulopathy; elevation of liver transaminases over twice the upper limit of the normal range; significant elevation of conjugated bilirubin [21].

During treatment with either of the two drugs, continuous monitoring of oxygen saturation, heart rate, respiratory rate, and blood pressure was performed using multi-parameter monitoring equipment. To investigate possible side effects of the drugs, the following tests were performed on each patient prior to treatment: serum creatinine, blood urea nitrogen, urine output measurement, AST, ALT, prothrombin time, activated partial thromboplastin, bilirubin, and blood count. These tests were repeated at 24 and 72 h after the start of treatment. In addition, a brain ultrasound was performed before and after treatment to rule out intracranial hemorrhage.

Primary PDA closure was considered when it occurred shortly after the first course of pharmacological treatment through an echocardiogram performed at the end of the third day of treatment. In case of persistence of the ductus, a second course of treatment was given using the same therapeutic scheme as earlier. Secondary closure was considered when it occurred after the second course of pharmacological treatment. In case of failure of ductal closure with the second course, most infants underwent surgical ligation.

### Statistical analysis

The study sample was obtained from a population of 239 VLBW newborns that met the inclusion criteria for this study. The sample size estimate was based on the literature information related to the administration of acetaminophen and ibuprofen in previous studies.

The sample size calculation was based on the expected hs-PDA closure rate proportions reported by Dani et al. [22]. Assuming an alpha error of 0.05, a test power of 0.8, and incorporating a 15% anticipated attrition rate (due to death, abandonment of treatment, among others), a total of 118 newborns was required. Ultimately, 120 neonates were randomized in a 1:1 ratio between the study groups.

For the storage of the information, a database was created in Microsoft Excel, to be later analyzed using the statistical program SPSS v. 23.

The qualitative variables were presented as absolute frequency and percentage relative frequency, while quantitative variables are presented as mean, standard deviation, minimum and maximum. Student's *t-*test was used to compare means, and Chi^2^ to compare proportions. A *p* value of < 0.05 was considered significant.

This study was approved by the Scientific and Ethics Committee of the Concepción Health Service and informed consent for each patient was obtained from one of the parents.

## Results

**Figure 1:**
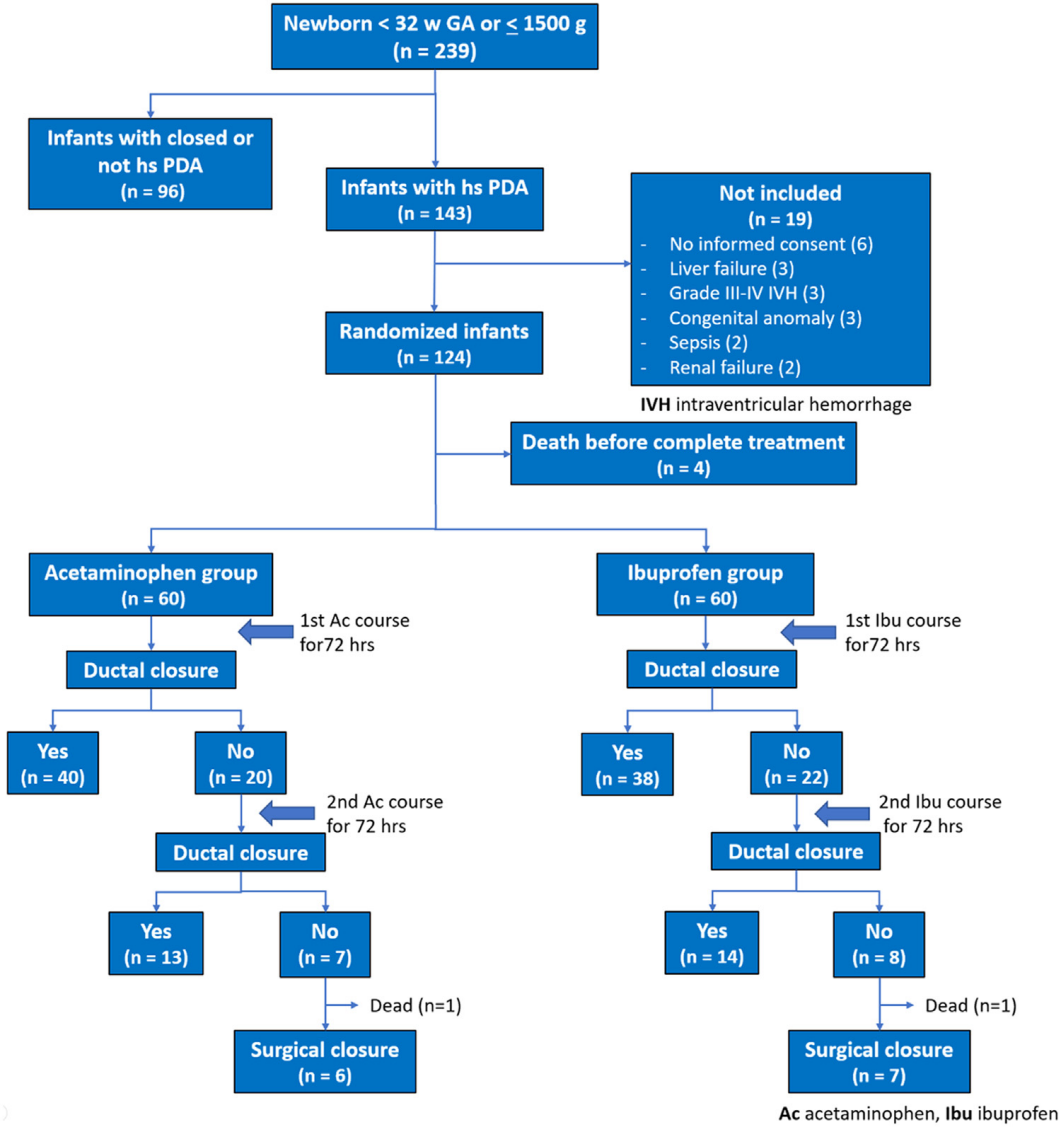
Randomization flowchart and study outcome.

120 preterm newborns who met the inclusion criteria were enrolled; 60 were randomized to the acetaminophen group and 60 to the ibuprofen treatment group (Figure 1). The mean weight and GA ± SD for the acetaminophen group were 1145 ± 339 g and 28.3 ± 2.5 weeks, and 1096 ± 251 g and 27.6 ± 2.1 weeks for the ibuprofen group (Table 1).

**Table 1:** Baseline demographic and clinical characteristics by assigned treatment group.

Variables	Acetaminophen group (*n* = 60)	Ibuprofen group (*n* = 60)	*p*
**Gestational age (weeks), x̄ ± SD**	28.3 ± 2.5	27.6 ± 2.1	0.09
**Birth weight (grams), x̄ ± SD**	1145 ± 339	1096 ± 251	0.39
**Male gender, *n* (%)**	36 (60.0)	35 (58.3)	0.60
**Cesarean delivery, *n* (%)**	43 (71.6)	36 (60.0)	0.18
**Antenatal steroids, *n* (%)**	52 (86.7)	49 (81.7)	0.32
**PROM > 18h, *n* (%)**	17 (28.3)	10 (16.7)	0.13
**Apgar Minute 1, median**	5	5	0.42
**Apgar Minute 5, median**	7	7	0.37
**FiO₂ (%), x̄ ± SD**	33.9 ± 13.3	32.7 ± 10.9	0.55
**Invasive mechanical ventilation, *n* (%)**	33 (55.0)	32 (53.3)	0.89
**Non-invasive respiratory support, *n* (%)**	36 (60.0)	32 (53.3)	0.57
**Surfactant for RDS, *n* (%)**	49 (81.7)	52 (86.7)	0.45
**Ductus arteriosus size (mm), x̄ ± SD**	2.35 ± 0.45	2.41 ± 0.50	0.40
**Newborn age at randomization (hours), x̄ ± SD**	49.8 ± 18.9	55.0 ± 28.3	0.27

PROM, premature rupture of membranes; RDS, Respiratory distress syndrome; FiO_2_, Fraction of inspired oxygen.

X^2^ test.

The average pre-treatment ductal diameter was similar between both groups (acetaminophen 2.35 mm and ibuprofen 2.41 mm). The age at the time of starting treatment was 49.8 ± 18.9 h in the acetaminophen group and 55.0 ± 28.3 h in the ibuprofen group (*p* = 0.27). Table 2 shows the rate of ductal closure after the first course of treatment, 66.7% (40/60) in the acetaminophen group and 63.3% (38/60) in the ibuprofen group (*p* = 0.70), increasing to 88.3% and 86.7%, respectively, after the second treatment course (*p* = 0.78). Surgical closure was required in 10% of the acetaminophen group (6/60) and 11.7% of the ibuprofen group (7/60; *p* = 0.77) (Table 2).

**Table 2:** Primary outcomes of neonates according to treatment group.

Primary outcome	Acetaminophen group (*n* = 60)	Ibuprofen group (*n* = 60)	*p*
**Primary ductal closure, *n* (%)**	40 (66.7)	38 (63.3)	0.70
**Secondary ductal closure, *n* (%)**	53 (88.3)	52 (86.7)	0.78
**Surgical closure, *n* (%)**	6 (10)	7 (11.7)	0.77

X^2^ test.

When analyzing the results according to subgroups by gestational age < and/or ≥ 28 weeks and birth weight < and/or ≥ 1000 g, infants weighing <1000 g had a tendency for lower primary closure in the acetaminophen vs ibuprofen group (36.8 vs 53.8%) (Table 3). This trend toward less primary ductal closure was also observed in infants <28 weeks of gestational age (Table 3). In both treatment groups, the infants who required surgical closure weighed <1000 g at birth and had a gestational age of <28 weeks, with the exception of one newborn in the group treated with acetaminophen (Table 3).

**Table 3:** Subgroup analyses of outcomes according to gestational age and birth weight.

Subgroups	Acetaminophen	Ibuprofen	*p*
**< 1000 g**	***n* = 19**	***n* = 26**	
Primary ductal closure, *n* (%)	7 (36.8)	14 (53.8)	0.26
Secondary ductal closure, ***n*** (%)	13 (68.4)	18 (69.2)	0.95
Surgical closure, *n* (%)	5 (26.3)	7 (26.9)	0.96
**≥ 1000 g**	*n***= 41**	*n***= 34**	
Primary ductal closure, *n* (%)	33 (80.5)	24 (70.6)	0.32
Secondary ductal closure, *n* (%)	40 (97.6)	34 (100)	0.36
Surgical closure, *n* (%)	1 (0.02)	0 (0)	0.36
**< 28 weeks**	***n* = 19**	***n* = 30**	
Primary ductal closure, ***n*** (%)	7 (36.8)	14 (46.7)	0.50
Secondary ductal closure, ***n*** (%)	13 (68.4)	22 (73.3)	0.71
Surgical closure, ***n*** (%)	5 (26.3)	7 (23.3)	0.81
**≥ 28 weeks**	***n* = 41**	***n* = 30**	
Primary ductal closure, ***n*** (%)	33 (80.5)	24 (80)	0.96
Secondary ductal closure, ***n*** (%)	40 (97.6)	30 (100)	0.39
Surgical closure, ***n*** (%)	1 (0.02)	0 (0)	0.39

X^2^ test.

No differences were detected in the most relevant morbidities such as intraventricular hemorrhage (*p* = 0.09), early and late onset sepsis (*p* = 0.81 and *p* = 0.26, respectively), necrotizing enterocolitis (*p* = 0.75), bronchopulmonary dysplasia (*p* = 0.46), retinopathy of prematurity (*p* = 0.28), and acute kidney injury (*p* = 0.82) (Table 4). Regarding retinopathy of prematurity, 4 patients progressed to the threshold stage in the acetaminophen group and 5 patients in the ibuprofen group, requiring treatment with bevacizumab (Avastin). There were no significant differences between the laboratory tests before and after treatment in any of the groups (Table 5). There were two deaths, one in every group, in the entire study population. Two cases of upper gastrointestinal bleeding were diagnosed in the ibuprofen group (*p* = 0.48).

**Table 4:** Secondary outcomes of neonates according to treatment group.

Secondary outcome	Acetaminophen group (***n*** = 60)	Ibuprofen group (***n*** = 60)	*p*
**RDS, *n* (%)**	57 (95)	58 (96.7)	0.65
**IVH grade 1–2, *n* (%)**	12 (20.0)	6 (10.0)	0.13
**IVH grade 3–4, *n* (%)**	7 (11.7)	5 (8.33)	0.54
**Pulmonary hemorrhage, *n* (%)**	5 (8.3)	6 (10.0)	0.75
**EOS, *n* (%)**	10 (16.7)	11 (18.3)	0.81
**AKI, *n* (%)**	12 (20)	11 (18.3)	0.82
**NEC, *n* (%)**	5 (8.3)	6 (10.0)	0.75
**GI bleeding, *n* (%)**	0 (0.0)	2 (3.3)	0.48
**Pneumonia, *n* (%)**	9 (15)	5 (8.3)	0.25
**LOS, *n* (%)**	21 (35.0)	27 (45.0)	0.26
**ROP, *n* (%)**	12 (20.0)	17 (28.3)	0.28
**BPD, *n* (%)**	26 (43.3)	30 (50.0)	0.46

RDS, respiratory distress syndrome; IVH, intraventricular hemorrhage; EOS, early onset sepsis; AKI, acute kidney injury; NEC, necrotizing enterocolitis; GI, gastrointestinal; LOS, late onset sepsis; ROP, retinopathy of prematurity; BPD, broncopulmonary dysplasia.

X^2^ test.

**Table 5:** Comparison of laboratory tests pre and post treatment in acetaminophen and ibuprofen group.

	Acetaminophen group, x̄ ± SD			Ibuprofen group, x̄ ± SD		
	**Pre**	**Post**	*p*	**Pre**	**Post**	*p*
**Serum creatinine (mg/dL)**	0.67 ± 0.27	0.64 ± 0.24	0.23	0.62 ± 0.31	0.59 ± 0.27	0.36
**Serum BUN (mg/dL)**	20.4 ± 8.9	21.8 ± 11.36	0.06	21.2 ± 9.4	23.4 ± 11.23	0,08
**Serum Bilirrubin level (mg/dL)**	6.4 ± 2,1	6.1 ± 2,1	0.23	6.4 ± 2,0	6.2 ± 1,8	0.36
**Serum ALT level (U/L)**	10.8 ± 5.5	11.4 ± 5.1	0.23	10.2 ± 5.2	11.1 ± 5.4	0.17
**Serum AST level (U/L)**	30.4 ± 14.9	26.5 ± 16.6	0.18	30.8 ± 15.4	25.3 ± 17.01	0.07
**Platelet count (x uL)**	171,317 ± 74,256	185,800 ± 104,109	0.22	171,666 ± 82,915	189,050 ± 108,985	0.09
**Serum Hb level (g/dL)**	13.0 ± 2.6	12.6 ± 2.0	0.13	12.4 ± 2.7	12.1 ± 2.0	0.20

BUN, blood urea nitrogen; ALT, alanine amino transferase; AST, aspartate amino transferase.

X^2^ test.

## Discussion

Since the first report by Hammerman et al. [23]. published more than a decade ago, the evidence on the use of acetaminophen and its advantages over other pharmacological alternatives for the closure of hs-PDA has been limited and, in many cases, inconsistent [24,25]

Furthermore, the general necessity of actively treating hs-PDA is currently a subject of intense debate. A recent meta-analysis suggests that active pharmacological or surgical treatment of hs-PDA during the first two weeks of life may be associated with a higher incidence of death or moderate to severe bronchopulmonary dysplasia compared to an expectant management approach [2]. Nevertheless, when active pharmacological closure is deemed clinically necessary by the medical team to avoid pulmonary overperfusion in extremely low birth weight preterm infants, establishing the safest and most effective drug alternative remains crucial.

The present study showed a similar primary ductal closure rate between acetaminophen (66.7%) and ibuprofen (63.3%). This similarity in closure between both drugs was also observed in other studies, with a range of ductal closure between 71–82% with acetaminophen and 76–78% with ibuprofen [13,26,27]. The European multicenter collaborative study[22]. carried out in a population with a gestational age, birth weight, and characteristics of the ductus arteriosus similar to the present study, presented a lower rate of primary ductal closure with acetaminophen compared to ibuprofen (52 vs 78%; *p* = 0.026). However, the final outcomes of ductal closure were similar in both groups.

In the present trial, the rate of primary ductal closure was lower than that published in other studies [7,13,26,27], which report a closure rate between 71 and 80% for acetaminophen and between 76 and 80% for ibuprofen. It should be noted that the studies by Yang et al. [26] and Bagheri et al. [27] were conducted in a population with a larger birth weight and gestational age than the present study, and this can explain the higher closure rate [28,29].

Two meta-analyses that compare the effectiveness of paracetamol versus NSAIDs do not show differences in the PDA closure rates [30,31].

The authors observed that a second course of acetaminophen and ibuprofen was effective in closing hs-PDA refractory to the first treatment course (88.3% with acetaminophen and 86.7% with ibuprofen). This increase in the rate of ductal closure with a second course of these drugs has also been reported in other studies [5,7,22,27], and it can avoid the need for surgical closure.

A special consideration is the effect of these drugs in the most immature infants, specifically those with a gestational age lower than 28 weeks and a birth weight under 1000 g. When analyzing the data in these subgroups, it must be made clear that there was no statistically significant difference between the treatments (*p* = 0.50 and *p* = 0.26, respectively). However, the authors noted an observational trend toward a lower primary ductal closure rate in the group treated with acetaminophen compared to ibuprofen for both the < 28 weeks (36.8%vs 46.7%) and < 1000 g (36.8% vs 53.8%) subgroups. These observational results are similar to those reported in the secondary analysis of the PDA-TOLERATE Trial [32]. However, the RCT reported by El-Mashad et al. [7]. in a population < 28 weeks gestational age found no difference in the rates of primary ductal closure between acetaminophen and ibuprofen [7]

A recent network meta-analysis between oral versus intravenous paracetamol suggests that oral paracetamol increases the rate of ductal closure when compared to IV paracetamol [33]

In the present study, the authors found no significant differences in adverse effects between both drugs — such as acute kidney injury (20.0% vs 18.3%, *p* = 0.82), necrotizing enterocolitis (8.3%vs 10.0%, *p* = 0.75), or gastrointestinal bleeding (0.0% vs 3.3%, *p* = 0.48)—as reported in previous clinical trials [13,22,26,34]. However, there are some reports showing differences in adverse effects between acetaminophen and ibuprofen [5,7,27]. It is worth mentioning that in 2 of these studies [5,27]. acetaminophen was administered orally, which could explain the lower side effects, possibly related to differences in route-specific pharmacokinetics, systemic exposure, study populations, and baseline risk; however, this explanation remains speculative.

A potential concern with the use of acetaminophen in VLBW infants is the possible alteration of liver function [31], something that was not observed in this study (ALT *p* = 0.23; AST *p* = 0.18), nor in other studies using a standard dose of acetaminophen (15 mg/kg/ dose every 6 h) [13,22,26,32,34].

### Strengths

The present study included a substantial number of very low birth weight neonates, compared to previous randomized studies. All cardiac evaluations were performed throughout the study period, with serial echocardiography done by the same two pediatric cardiologists, providing consistent evaluation of the ultrasonographic parameters that defined a hs-PDA.

In addition, the study was carried out with the same ductal diagnostic and treatment criteria, and also the same medical and nursing staff during the 3 years of the study.

### Weaknesses

The study was not completely blinded because the dose and schedule of the drugs administered were different. This partial blinding could potentially introduce performance bias; however, to mitigate its impact on the interpretation of the results, it was blinded for the pediatric cardiologists who performed the echocardiography and also for the health personnel who cared for the patients.

The number of infants <28 weeks' gestational age, who are the most interesting group to study, was relatively small (***n*** = 49, representing 40.8% of the total cohort), which limits the statistical power to draw definitive conclusions for this specific subgroup.

## Conclusion

Acetaminophen may be considered a safe and effective alternative, with no differences in side effects, to ibuprofen for the treatment of hs-PDA in very low birth weight premature infants.

## Trial registration number

ISRCTN72579770

## Funding

This research did not receive any specific grant from funding agencies in the public, commercial, or not-for-profit sectors.

## Data availability

The clinical datasets generated and analyzed during the current study are not publicly available in open online repositories due to strict patient confidentiality policies and ethical restrictions established by the Scientific and Ethics Committee of the Concepción Health Service and Guillermo Grant Benavente Hospital, but are available from the corresponding author on reasonable request.

## Conflicts of interest

The authors declare no conflicts of interest.
